# One Screening Magnetic Resonance Imaging Sequence in Evaluation of Chondral and Meniscal Lesions of the Knee − A Pilot Study

**DOI:** 10.2174/1874325000802010019

**Published:** 2008-02-15

**Authors:** Paavo-Ilari Kuikka, Ole M Böstman, Martti J Kiuru, Sari T Salminen, Sakari Mikkola, Harri K Pihlajamäki

**Affiliations:** 1 Centre for Military Medicine, P.O. Box 50, FIN-00301 Helsinki, Finland; 2 Department of Orthopaedic and Trauma Surgery, Helsinki University Central Hospital, Helsinki, Finland; 3 Department of Radiology, Helsinki University Central Hospital, Helsinki, Finland; 4 Orton Invalid Foundation, Helsinki, Finland; 5 Department of Pediatric Surgery, Helsinki University Central Hospital, Helsinki, Finland; 6 Department of Surgery, Central Military Hospital, Helsinki, Finland

**Keywords:** Osteoarthritis, knee, articular cartilage, MRI, arthroscopy.

## Abstract

This prospective study aimed to evaluate if chondral and meniscal lesions in symptomatic knees of osteoarthritis patients can be reliably identified using only one sagittal dual-echo MRI (Magnetic Resonance Imaging) sequence. MRI was performed on 13 patients after knee arthroscopy due to knee pain and clinically suspected osteoarthritis using a 1.5-Tesla scanner with knee coil and a sagittal dual-echo turbo spin-echo PD (Proton Density)- and T2-weighted sequence. The MRI and arthroscopic findings were then compared. Of 65 articular surfaces, 47 were damaged. For articular cartilage lesions, the overall sensitivity of MRI was 46.8%, specificity 72.2%, and diagnostic accuracy 53.9%, and for meniscal ruptures 81.2%, 66.7%, and 73.1%, respectively. The present study showed that the reliability of screening MRI of knees using only one sagittal dual-echo sequence does not suffice for diagnosis of chondral or meniscal lesions, and should therefore not replace routine knee MRI or diagnostic arthroscopy.

## INTRODUCTION

The majority of internal knee derangements in osteoarthritis patients consist of chondral and meniscal lesions. In order to evaluate the need for an arthroscopic operation, MRI examination of the knee joint is often performed. According to previous research, MRI offers good sensitivity and specificity for examining menisci and ligament injuries of the knee [1-7]. With the 1.5 Tesla MRI sequences chondral lesions can also be detected reliably [8-12].

In routine knee MRI, sagittal PD sequence is mainly used to evaluate meniscal and ligamentous problems of the knee and T2-weighted sequence can be used to detect chondral lesions. The diagnosis is normally ensured and reinforced with several imaging planes and sequences. A routine knee MRI usually consists of four to six sequences. In the case that only one screening sequence would be reliable enough for detecting internal derangements of the knee, faster and cheaper MRI examination of the knee joint would be possible.

The purpose of this prospective study was to evaluate if chondral and meniscal lesions in a symptomatic knee of an osteoarthritis patient can be detected reliably with only one sagittal dual-echo MRI sequence.

## MATERIALS AND METHODOLOGY

For the purposes of this prospective study, MRI examination of the knee was performed on patients before knee arthroscopy at the University Central Hospital of our district.

The inclusion criteria for the present study consisted of an age over 45 years, a long-term (more than six months) pain in or swelling of the knee joint and clinically suspected osteoarthritis. All patients included in the study were chosen for arthroscopy of the knee according to the normal policy of the department. The exclusion criteria for the study consisted of a posttraumatic osteoarthritis and a fresh (less than 6 months) trauma of the knee region. Using the aforementioned criteria 13 patients were selected for MRI before arthroscopy of the knee for the purposes of this series. Seven of the patients were men and six were women. The age range varied from 46 to 70 years and the mean age was 53.8 years.

All patients underwent a 1.5 Tesla MRI (Magnetom Vision; Siemens Medical Systems, Iselin NJ, Erlangen, Germany) examination. The MRI screening examination was performed with a knee coil and a sagittal dual-echo turbo spin-echo PD- and T2-weighted sequence (TR/TE 2500/16 and 98, FA 180ْ, FOV 160 x 160 mm, matrix 220 x 256, the section thickness was 3 mm, gap 1 mm, number of slices 20, and with one signal average) was used.

MR images were evaluated by a musculoskeletal radiologist. Five articular surfaces were evaluated in each knee: patella, medial and lateral femoral condyle, and medial and lateral tibial plateaus. Lateral and medial menisci of the knees were also evaluated and interpreted. The arthroscopy was performed within three months of the MRI. While performing the arthroscopy, the orthopaedic surgeon evaluated the cartilage lesions and meniscal ruptures without knowledge of the MRI findings.

A numerical grading system developed by Tyrrell *et al*. [13], based on the depth of the chondral lesion, was employed in the categorisation of results from MRI and arthroscopy (Table **1**). For calculation of specificity, sensitivity, and diagnostic accuracy with single table analysis, results from arthroscopy were used as the gold standard. Ninety-five percent confidence intervals were calculated with Wilson Score method [14].

## RESULTS

Using MRI and arthroscopy, a total of 65 articular cartilage surfaces in 13 knees were assessed. In arthroscopy, 18 of these surfaces were found to be intact and normal, 21 were determined to be Grade I, 13 were Grade II, and 13 were Grade III. The number of articular surfaces with lesions totalled 47.

On MRI, cartilage damages were discovered with an overall sensitivity of 46.8% (95% confidence interval 33.3-60.8), specificity of 72.2% (95% confidence interval 49.1-87.5) and diagnostic accuracy of 53.9% (95% confidence interval 41.9-65.4) while arthroscopy was considered the gold standard. Grade I lesions were detected with a sensitivity and diagnostic accuracy of only 28.6% (95% confidence interval 13.8-50.0). Grade II lesions (Fig. **1A**,**B**) were detected with the sensitivity and diagnostic accuracy of 46.15% (95% confidence interval 23.2-70.9) and grade III lesions (Fig. **2A**,**B**) with the sensitivity and diagnostic accuracy of 76.9 (95% confidence interval 49.7-91.2).

In comparing the results between the two methods, there were 23 articular surfaces with identical appearance both on arthroscopy and on MRI. Differences were observed in 29 surfaces by one grade, in eight surfaces by two, and in five surfaces by three grades.

The chondral lesions were situated in various locations: twelve of the total 47 were found in the medial tibial plateau, 11 in the lateral tibial plateau, nine in the medial femoral condyle, eight in the lateral femoral condyle, and seven lesions in the patellar surface. Grades of the lesions and MRI results in different articular surfaces are presented in Tables **2** and **3**.

Lesions in multiple articular surfaces were found in eleven patients and a concominant meniscal rupture was found in nine patients.

Rupture of the medial menisci was detected in eight and rupture of the lateral menisci in three knees. All three knees with rupture of a lateral meniscus also included rupture of a medial meniscus. There were four knees without internal derangements (ligament or meniscal injuries) other than the chondral lesions. MRI detected meniscal ruptures with the sensitivity of 81.2% (95% confidence interval 52.3-94.9), specificity of 66.7% (95% confidence interval 41.7-84.2), and diagnostic accuracy of 73.1% (95% confidence interval 53.9-86.3).

## DISCUSSION

According to our results, it appears that only one sagittal screening MRI sequence is not reliable enough to be used as a diagnostic tool for chondral and meniscal injuries. It seems that it is not reasonable to decrease the number of sequences used in a routine knee MRI in order to reduce the expenses and make the examination less time-consuming.

At best, sequences in common clinical use today are very reliable. Examining 130 patients, Bredella *et al*. [8] used 1.5 Tesla magnet with a combination of all three planes, sagittal, coronal, and axial. They were able to discover cartilage abnormalities with a sensitivity of 93%, specificity of 99%, and accuracy of 98%. In their research, arthroscopy was likewise considered the gold standard. Systematic review by MacKenzie *et al*. [15] included 7 studies that reported overall sensitivity of 89%, spesificity of 92% and accuracy of 91% for lateral and medial meniscal ruptures. Total number of menisci examined was 982.

In the present study, a dual-echo sequence was used for the sagittal screening sequence. The dual-echo sequence is a sequence where two sequences (PD and T2) are obtained simultaneously without doubling the scanning time. PD is used to evaluate menisci lesions and T2-weighted for chondral evaluation. In normal routine knee MRI, the menisci evaluation is mainly done from the sagittal plane images, although other planes, mainly coronal, are used to reinforce the interpretation.

The lack of other imaging planes affected least to the sensitivity of menisci lesions in the present study but better results have been achieved widely [1-3, 5-7]. However, in the present study, the PD sequence was a turbo spin-echo sequence, which in a recently published study [16] was shown to have an approximately 10% lower sensitivity than conventional spin-echo PD in menisci tears (80 % *vs* 93%). The impairment of the specificity was clearer and approximately 25% lower than the average result of previously published studies [15]. Impairment of diagnostic accuracy was also clear. In the evaluation of chondral lesions the lack of other imaging planes, axial for patellofemoral and coronal for tibiofemoral, clearly decreased the MRI sensitivity, specificity and diagnostic accuracy for chondral lesions when compared to previous studies [8-12].

In the present study MRI sensitivity in chondral lesions varied depending on the grade of the chondral lesion. The decrease in sensitivity was seen especially in lower grade (more superficial) chondral lesions, where the MRI changes are more subtle and an optimal imaging plane is essential for their detection. In higher grade lesions affecting the subchondral bone, the sensitivity increased but even within these chondral lesions the lack of other imaging planes decreased the sensitivity when compared to previous studies [8]. It is clinically more important to detect especially these deep lesions because they may cause symptoms and pain to the patient. Confusingly, there is evidence suggesting that it may not always be advantageous to perform arthroscopic lavage or debridement for osteoarthritis knees at all [17].

The significance of different imaging planes in MRI was also shown as there was a clear difference between MRI results in different articular surfaces. However, also the grades of the lesions varied in different articular surfaces and can explain the result. For example, medial femoral condyle in which the MRI results were most reliable, included several grade III lesions and only one grade I lesion. Lateral tibial plateau, on the other hand, included seven grade I lesions and only two grade III lesions and MRI results were poor for that reason.

The limitation of the study was the small number of patients examined. However, it is reasonable to perform this kind of study at first with a small patient group before launching more comprehensive studies. Because of the unreliable results of MRI, it became clear that it is inappropriate to perform further studies with the methods used even though the number of patients in the present study was limited.

Arthroscopy is often referred to as the gold standard for non-invasive imaging studies of the knee, but not without some doubts as for its precision in the assessment of meniscal lesions [18]. We may agree with these claims considering the fact that arthroscopy allows only a visual inspection of the meniscal surface without access to the midsubstance of the tissue, possibly containing hidden lesions.

Even though MRI is nowadays reliable diagnostic tool, it is still not clear whether patients should undergo arthroscopy only after a complete primary MRI examination. For those in evident need of therapeutic arthroscopy, MRI can be considered as a misuse of resources. On the other hand, in cases where surgery is not relevant, arthroscopy should not be recommended either.

## CONCLUSION

In conclusion, the present study showed that the screening knee MRI with only one sagittal dual-echo sequence is not reliable enough to be used as a diagnostic tool for chondral or meniscal lesions and should not be used to replace routine knee MRI or diagnostic arthroscopy in a symptomatic knee. Considering the results of the present study, further studies of the topic using this study design seem to be inappropriate.

## Figures and Tables

**Fig. (1A):**
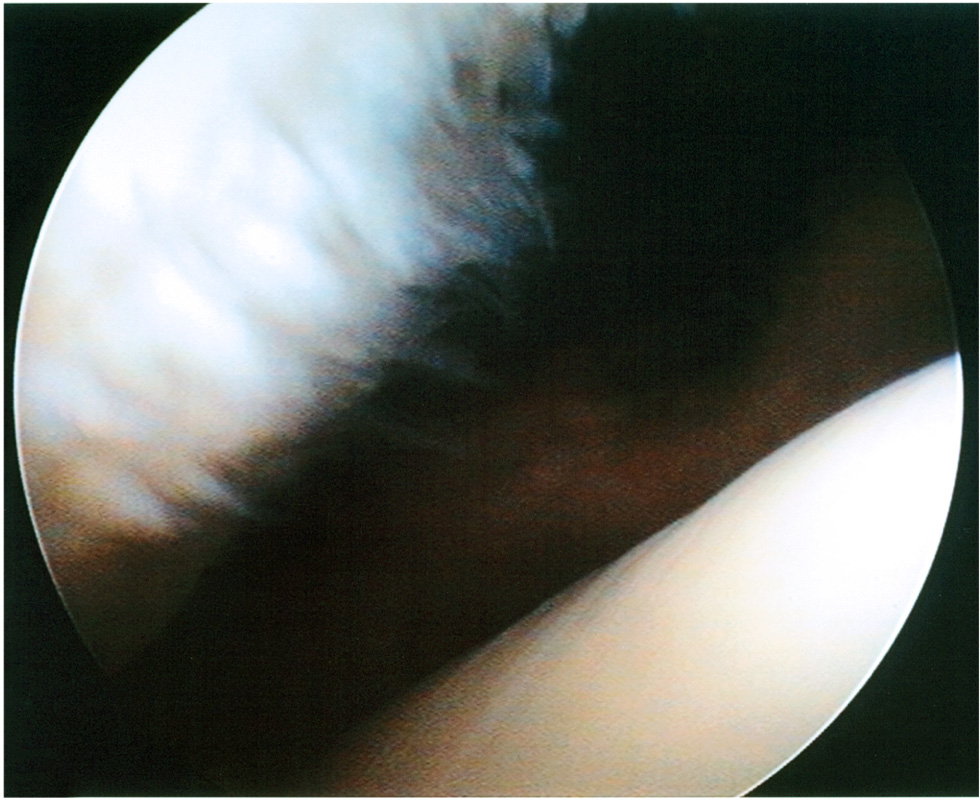
Arthroscopy reveals grade II lesion of the left patella in 49-year old woman. Patellar surface is clearly irregular but the subchondral bone is not exposed. Femoral surface seems to be normal and intact.

**Fig. (1B):**
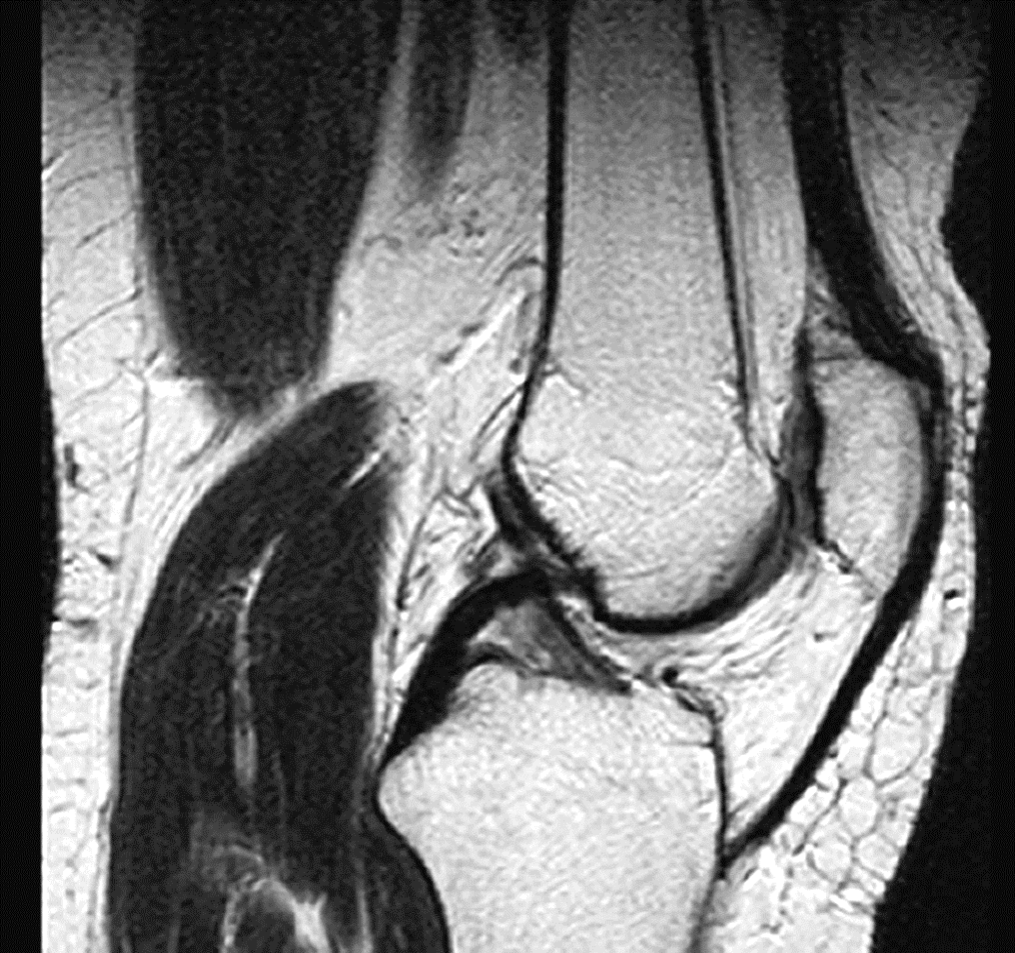
In magnetic resonance image, patellar surface lesion is interpreted to be grade II

**Fig. (2A):**
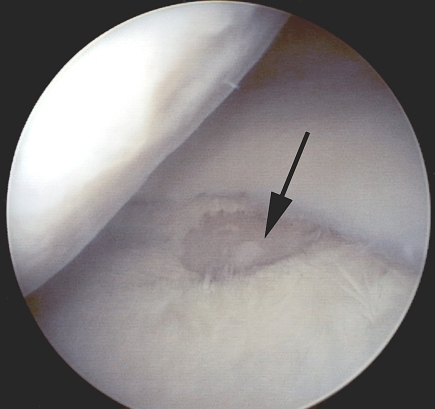
Arthroscopic view of grade III lesion of the medial tibial plateau of the right knee in 57-year old man. Subchondral bone is exposed (black arrow). Mild irregularity (grade I) can be seen in arthroscopy in medial femoral condyle.

**Fig. (2B):**
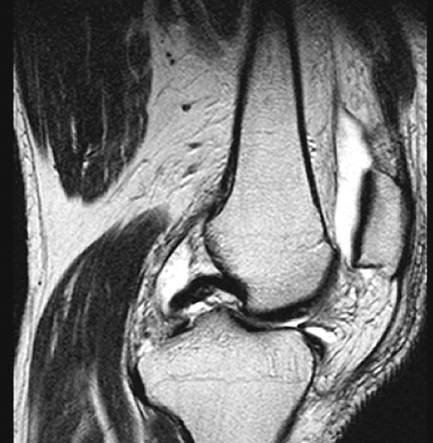
The grade III lesion of the medial tibial plateau in Fig. (2A) was interpreted to be grade II in magnetic resonance image. Grade I irregularity of the medial femoral condyle was not detected in MRI.

**Table 1:** Depth of Articular Cartilage Lesions According to the Grading Scale Developed by Tyrrell *et al.* [13]

Grade 0	normal
Grade 1	moderate irregularity
Grade 2	severe irregularity but not full thickness
Grade 3	full thickness loss

**Table 2:** Arthroscopic Grades and Locations of Chondral Lesions

Articular Surface	Arthroscopy Grade			
	0	I	II	III
Patella	6	3	4	0
Medial femoral condyle	4	1	2	6
Lateral femoral condyle	5	5	2	1
Medial tibial plateau	1	5	3	4
Lateral tibial plateau	2	7	2	2

**Table 3:** Reliability of Sagittal Magnetic Resonance Imaging Sequence in Different Articular Surfaces

Articular Surface	Variable	Value	95% CIs*
Patella	Sensitivity	57.1	25.5-84.2
	Specificity	50	18.8-81.2
	Diagnostic accuracy	53.9	29.1-76.8
Medial Femoral Condyle	Sensitivity	77.8	45.3-93.7
	Specificity	100	51.0-100
	Diagnostic accuracy	84.6	57.8-95.7
Lateral Femoral Condyle	Sensitivity	25	7.1-59.1
	Specificity	60	23.1-88.2
	Diagnostic accuracy	38.5	17.7-64.5
Medial tibial Plateau	Sensitivity	58.3	32.0-80.7
	Specificity	100	20.7-100
	Diagnostic accuracy	61.54	35.5-82.3
Lateral tibial Plateau	Sensitivity	18.2	5.1-47.7
	Specificity	100	34.2-100
	Diagnostic accuracy	30.8	12.7-57.6

* Confidence Intervals.
